# Supplementary material for: Mechanism of action for small-molecule inhibitors of triacylglycerol synthesis
Source: Nat Commun. 2023 May 29;14:3100. doi: 10.1038/s41467-023-38934-3 (PMC10227072; doi:10.1038/s41467-023-38934-3)
Supplement: Supplementary file 1 — Supplementary information [file 41467_2023_38934_MOESM1_ESM.pdf]

Supplementary Materials for

Mechanism of action for small-molecule inhibitors of triacylglycerol synthesis

Xuewu Sui[1,2,7], Kun Wang[1,2], Kangkang Song[3,4], Chen Xu[3,4], Jiunn Song[1,2], Chia-Wei Lee[1,2], Maofu Liao[2,8#], Robert V. Farese Jr.[1,2,5,9#] and Tobias C. Walther[1,2,5,6,9#]

Affiliations:
[1]Department of Molecular Metabolism, Harvard T.H. Chan School of Public Health, Boston, MA, USA.
[2]Department of Cell Biology, Harvard Medical School, Boston, MA, USA.
[3]Department of Biochemistry and Molecular Biotechnology, University of Massachusetts Chan Medical School, Worcester, MA, USA.
[4]Cryo-EM Core Facility, University of Massachusetts Chan Medical School, Worcester, MA, USA
[5]Broad Institute of MIT and Harvard, Cambridge, MA, USA.
[6]Howard Hughes Medical Institute, Boston, MA, USA.
[7]Department of Biochemistry and Biophysics, College of Agriculture and Life Sciences, Texas A&M University, TX, USA.
[8]School of Life Sciences, Southern University of Science and Technology, Shenzhen, China
[9]Cell Biology Program, Sloan Kettering Institute, Memorial Sloan Kettering Cancer Center, New York, NY, USA.

The PDF files includes
Supplementary Figures 1 to 10
Supplementary Table 1

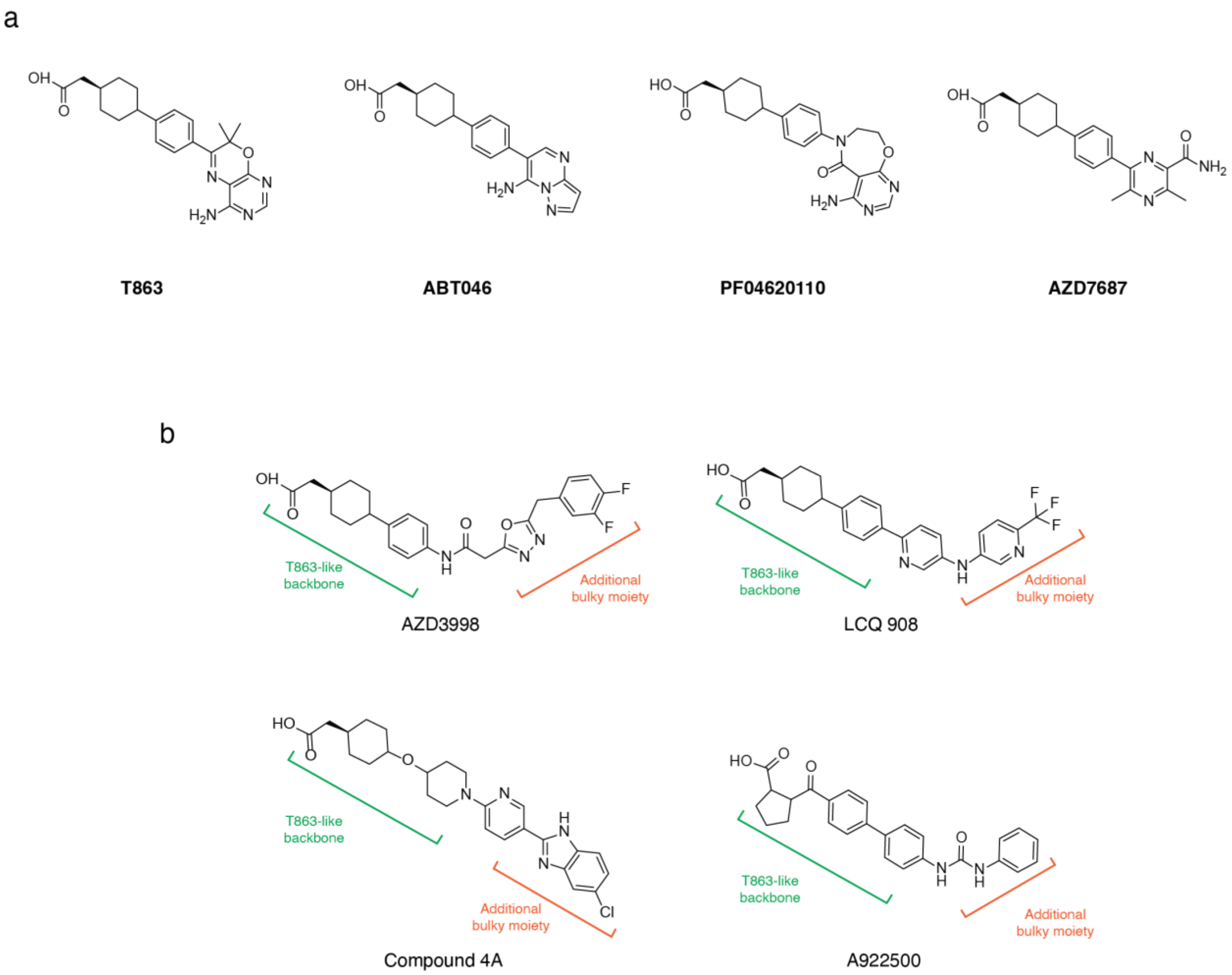

Supplementary Fig. 1 | Chemical structure of DGAT1 inhibitors. a, Examples of T863-like inhibitors that share a common chemical architecture with T863. **b,** Examples of T863-derived inhibitors. Note in addition to the T863-like moiety (green region), all contain an additional bulky moiety (orange region) attached to the T863-like backbone.

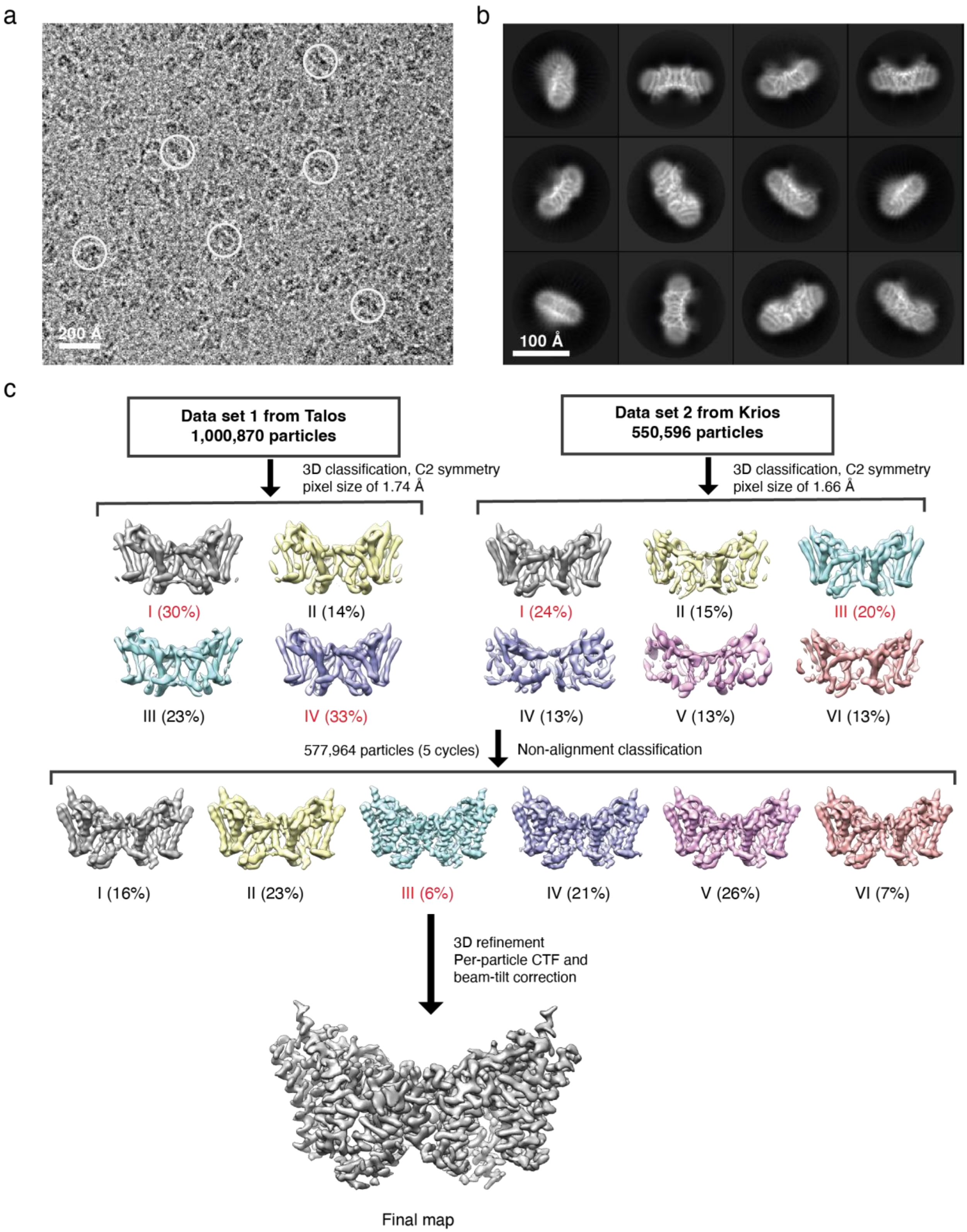

Supplementary Fig. 2 | Cryo-EM imaging processing of DGAT1-T863 complex. a, Representative cryo-EM images of DGAT1 with T863. The white circles outline several DGAT1 particles. **b**, The 2D class averages of cryo-EM particle images. **c**, Three-dimensional (3D)

classification and refinement of cryo-EM particles. Two cryo-EM data sets were collected and processed separately. After an initial round of 3D classification, particles within classes showing strong transmembrane densities (in red) in the final five iterations (indicated as '5 cycles') were kept and combined. The resulting selected particles were further classified by non-alignment classification. Afterwards, one class with highest estimated resolution and the best protein sidechain and T863 signals by visual inspection were used for application of a per-particle contrast transfer function (CTF) and beam-tilt corrections. The resulting refined cryo-EM map was used for model building and structural analysis.

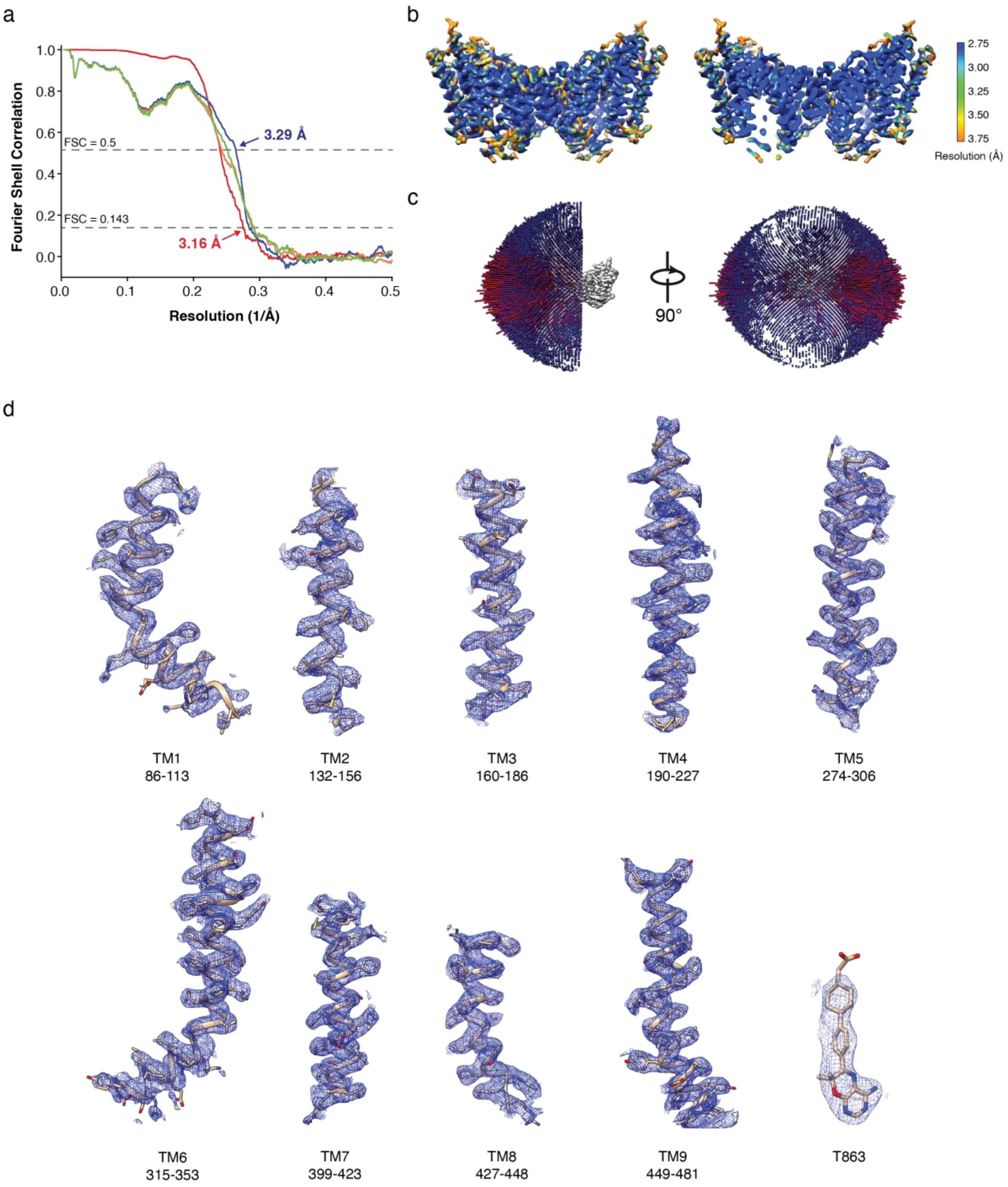

Supplementary Fig. 3 | Single-particle cryo-EM analysis of DGAT1-T863 complex. a, Fourier shell correlation (FSC) curves: gold-standard FSC curve between the two half maps with indicated resolution at FSC = 0.143 (red). The value (in red) was used for the final reported resolution; FSC curve between the atomic model and the final map with indicated resolution at FSC = 0.5 (blue);

FSC curve between half map 1 (orange) or half map 2 (green) and the atomic model refined against half map 1. **b**, Local resolution of the final cryo-EM map of DGAT1-T863 complex. A sliced view of local resolution is shown on the right. **c**, Cutaway view of angular distribution of particle images included in the final 3D reconstruction. **d**, Cryo-EM densities superimposed with atomic model for all nine transmembrane helices (TM1–TM9) and T863. Maps are contoured at 4–5σ.

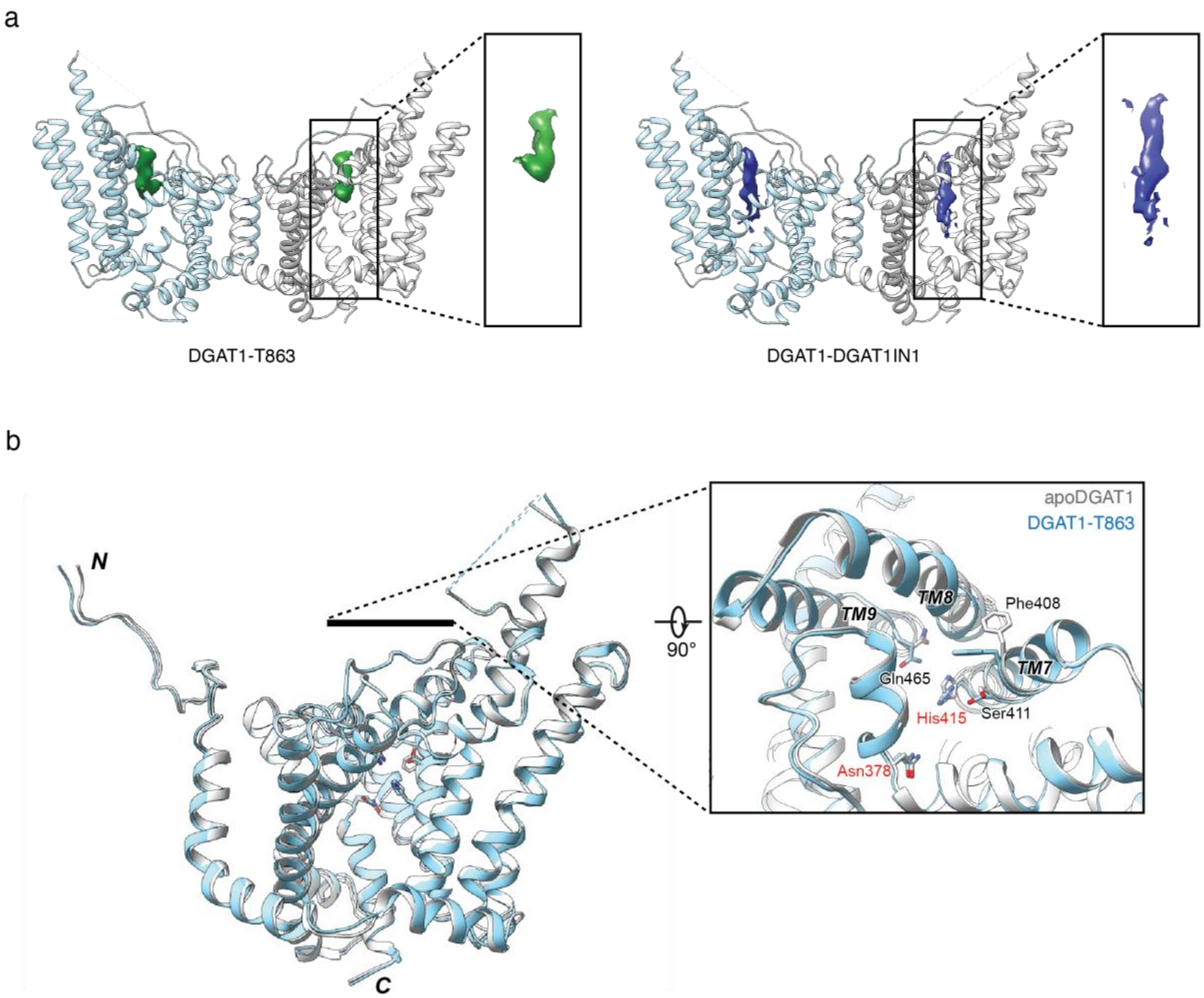

Supplementary Fig. 4 | Cryo-EM density and conformation analyses at the fatty acyl CoA binding site. a, A side-by-side comparison of the EM density at the fatty acyl CoA binding tunnel in T863-bound DGAT1 (DGAT1-T863) and DGAT1IN1-bound DGAT1 (DGAT1-DGAT1IN1). The EM maps in each state are contoured at the same level (3.5 σ). **b**, Conformational changes in the drug binding pocket between apo and T863 binding states. Note Phe^{408} and Gln^{465} adopt changed confirmations upon T863 binding. The catalytic residues His^{415} and Asn^{378} are labeled in red.

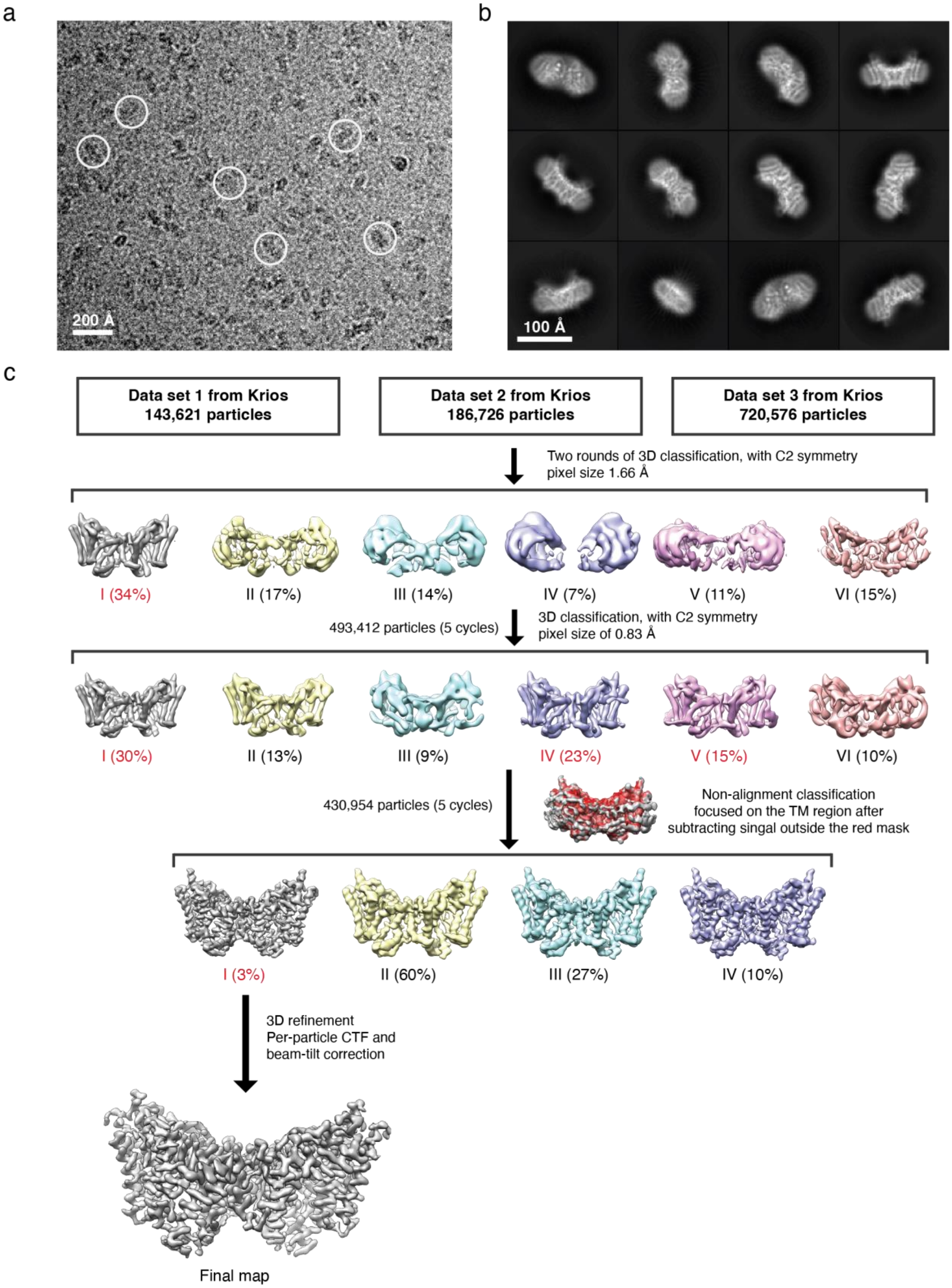
a
200 Å
b
100 Å
c
Data set 1 from Krios
143,621 particles
Data set 2 from Krios
186,726 particles
Data set 3 from Krios
720,576 particles
Two rounds of 3D classification, with C2 symmetry
pixel size 1.66 Å
I (34%)
II (17%)
III (14%)
IV (7%)
V (11%)
VI (15%)
493,412 particles (5 cycles)
3D classification, with C2 symmetry
pixel size of 0.83 Å
I (30%)
II (13%)
III (9%)
IV (23%)
V (15%)
VI (10%)
430,954 particles (5 cycles)
Non-alignment classification
focused on the TM region after
subtracting singal outside the red mask
I (3%)
II (60%)
III (27%)
IV (10%)
3D refinement
Per-particle CTF and
beam-tilt correction
Final map

Supplementary Fig. 5 | Cryo-EM imaging processing of DGAT1-DGAT1IN1 complex. a, Representative cryo-EM images of DGAT1 with DGAT1IN1. White circles outline some DGAT1 particles. **b**, 2D class averages of cryo-EM particle images. **c**, 3D classification and refinement of cryo-EM particles. Three cryo-EM data sets were combined for the first two round of 3D classification. Afterwards, class I that shows the strongest transmembrane densities (in red) in the final five iterations (indicated as '5 cycles') was selected for further classification. This classification steps generated three classes with the most abundant particles and strong transmembrane signal (in red), which were kept for further classification. The final classification step was performed by subtracting the micelle signal followed by non-alignment classification focusing on the EM signals within the red mask. Afterwards, class I exhibited the highest estimated resolution and the best protein sidechain and DGAT1IN1 signals by visual inspection and was used applying a per-particle CTF and beam-tilt corrections. The resulting refined cryo-EM map was used for model building and structural analysis.

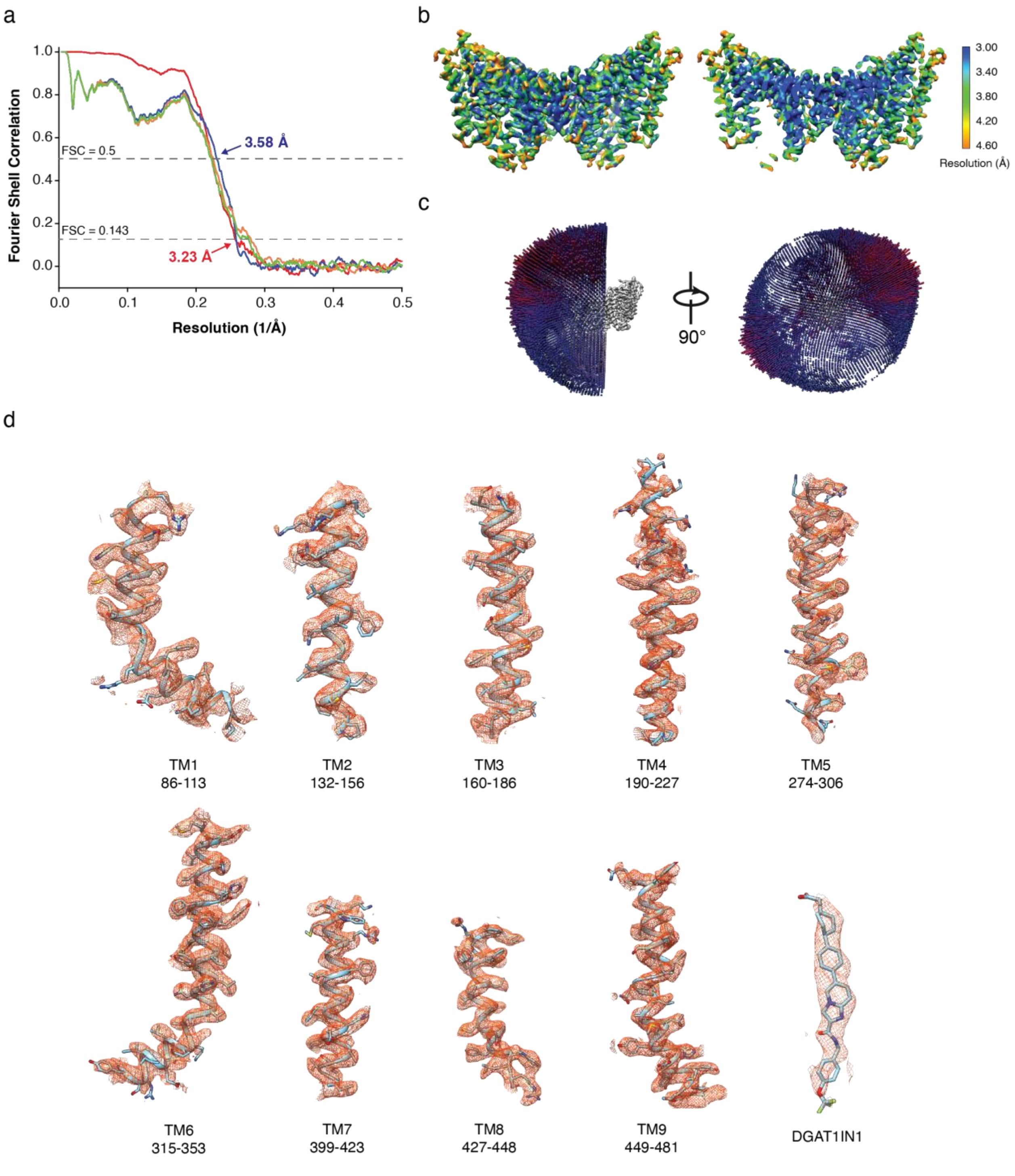

Supplementary Fig. 6 | Single-particle cryo-EM analysis of DGAT1 with DGAT1IN1. a, FSC curves: gold-standard FSC curve between the two half maps with indicated resolution at FSC = 0.143 (red). The value (in red) was used for the final reported resolution; FSC curve between the atomic model and the final map with indicated resolution at FSC = 0.5 (blue); FSC curves between half map 1 (orange) or half map 2 (green) and the atomic model refined against half map 1. **b**, Local resolution of the final cryo-EM map of DGAT1 with DGAT1IN1. A sliced view of local

resolution is shown on the right panel. **c**, Cutaway view of angular distribution of particle images included in the final 3D reconstruction. **d**, Cryo-EM densities superimposed with the atomic model for TM1-TM9 and DGAT1IN1. Maps are contoured at 4–5σ.

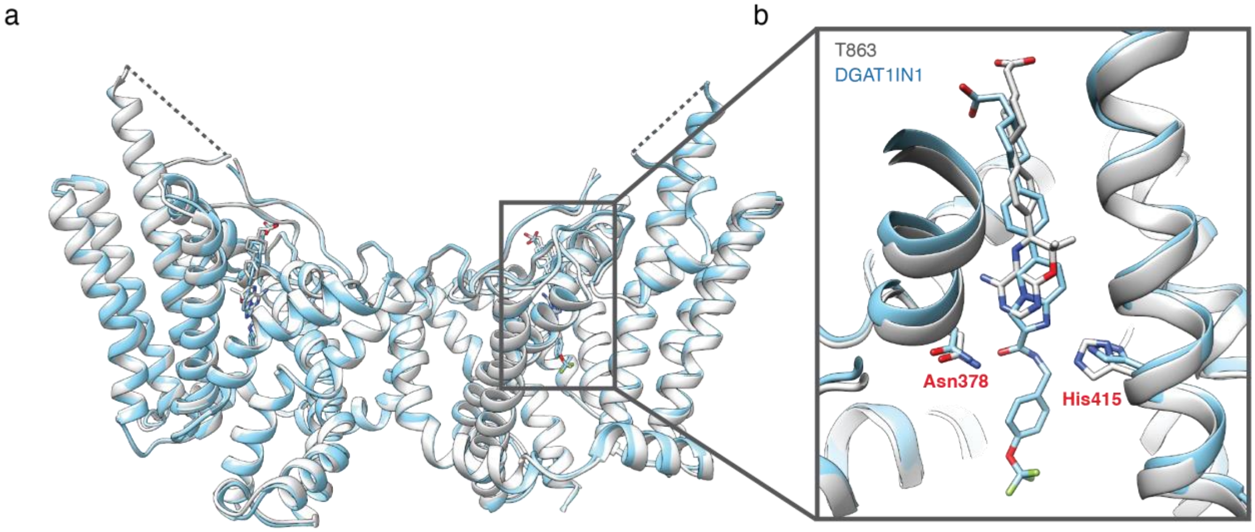

Supplementary Fig. 7 | Structural comparison of DGAT1 bound to T863 or DGAT1IN1. a, Ribbon representation of superimposed DGAT1-T863 and DGAT1-DGAT1IN1 complex structures. The Cα positions of 405 matched residues in each structure were superimposed with an RMSD of 0.511 Å. **b**, Zoomed-in view of the fatty acyl CoA binding tunnel bound to inhibitors. T863 and DGAT1IN1 are shown as gray and blue sticks, respectively. The catalytic residues of His^{415} and Asn^{378} are shown as sticks and labeled in red. DGAT1IN1 inserts into the fatty acyl-CoA binding tunnel approximately 1.2 Å deeper compared with T863.

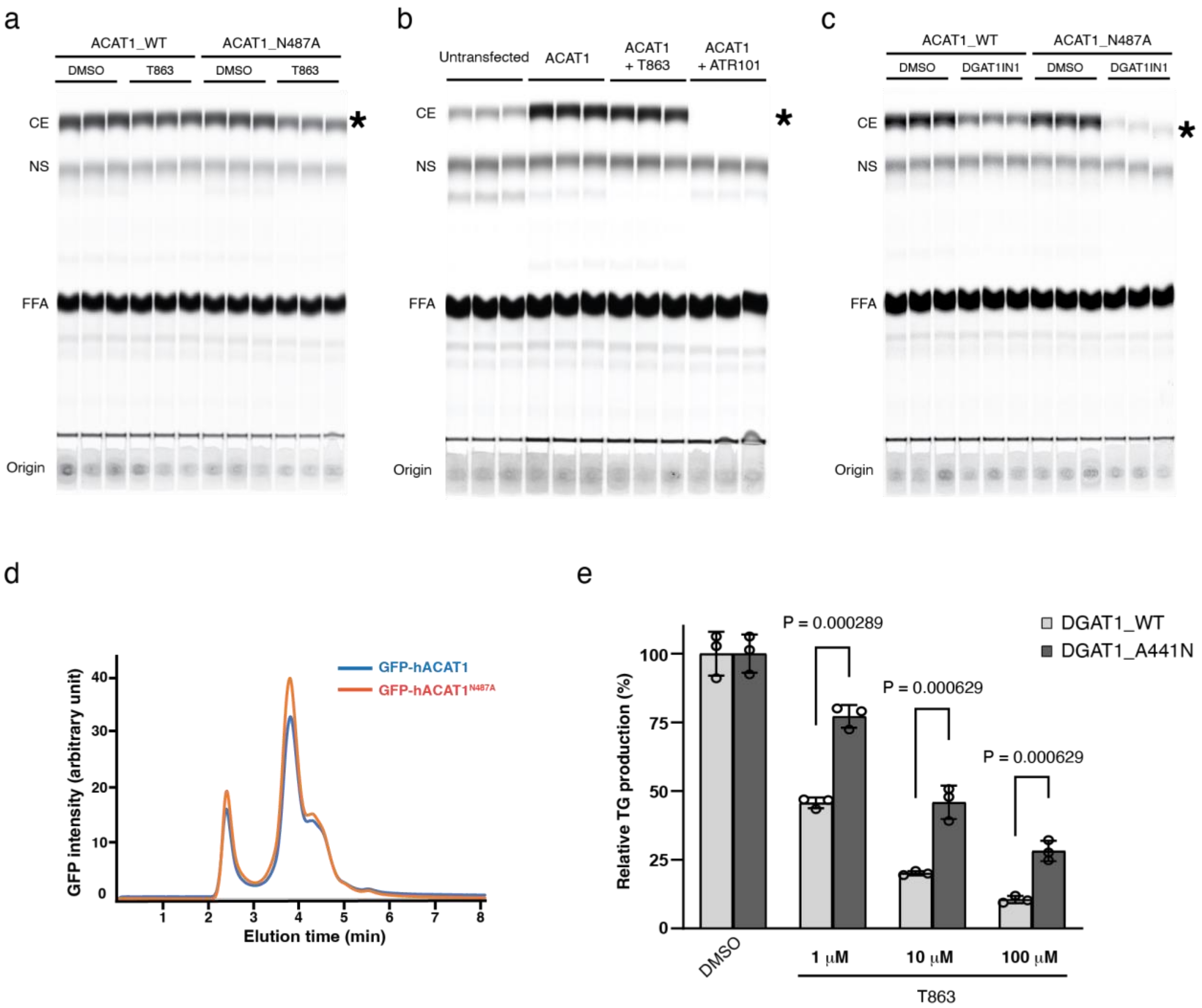

Supplementary Fig. 8 | Acyl-transferase activity analyses of human ACAT1 mutant. a-c, TLC of microsomal wild type ACAT or Asn487Ala mutant activity from regular or ACAT1 overexpression HEK293F cells in the presence of various inhibitors. Control HEK293F microsomes show endogenous ACAT activity in (**b**). All samples shown in (**a-c**) were loaded on a single TLC plate. The quantifications of ACAT1 reaction product on each TLC plate are shown in **Fig. 4c**,**d** and **f**, respectively. T863 and ATR101 were applied 10 µM and 1 µM, respectively. Mean ± s.d., n = 3 independent experiments. Experiments were repeated three time with similar results. FFA, free fatty acid; NS, non-specific band; ns, non-significant. **d**, Gel filtration profiles of wild type ACAT1 and the Asn487Ala mutant. The folding of ACAT1 and the mutant were analyzed by size-exclusion chromatography by monitoring GFP fluorescence. **e**, DGAT1 Ala441Asn mutation shows decreased T863 inhibition. Experiments were repeated three time with similar results. Data are shown from one representative result, and data points are shown as mean ± s.d., calculated from three technical replicates. Statistical analysis by multiple unpaired t tests two-sided analysis. Activities were normalized to DMSO treated control.

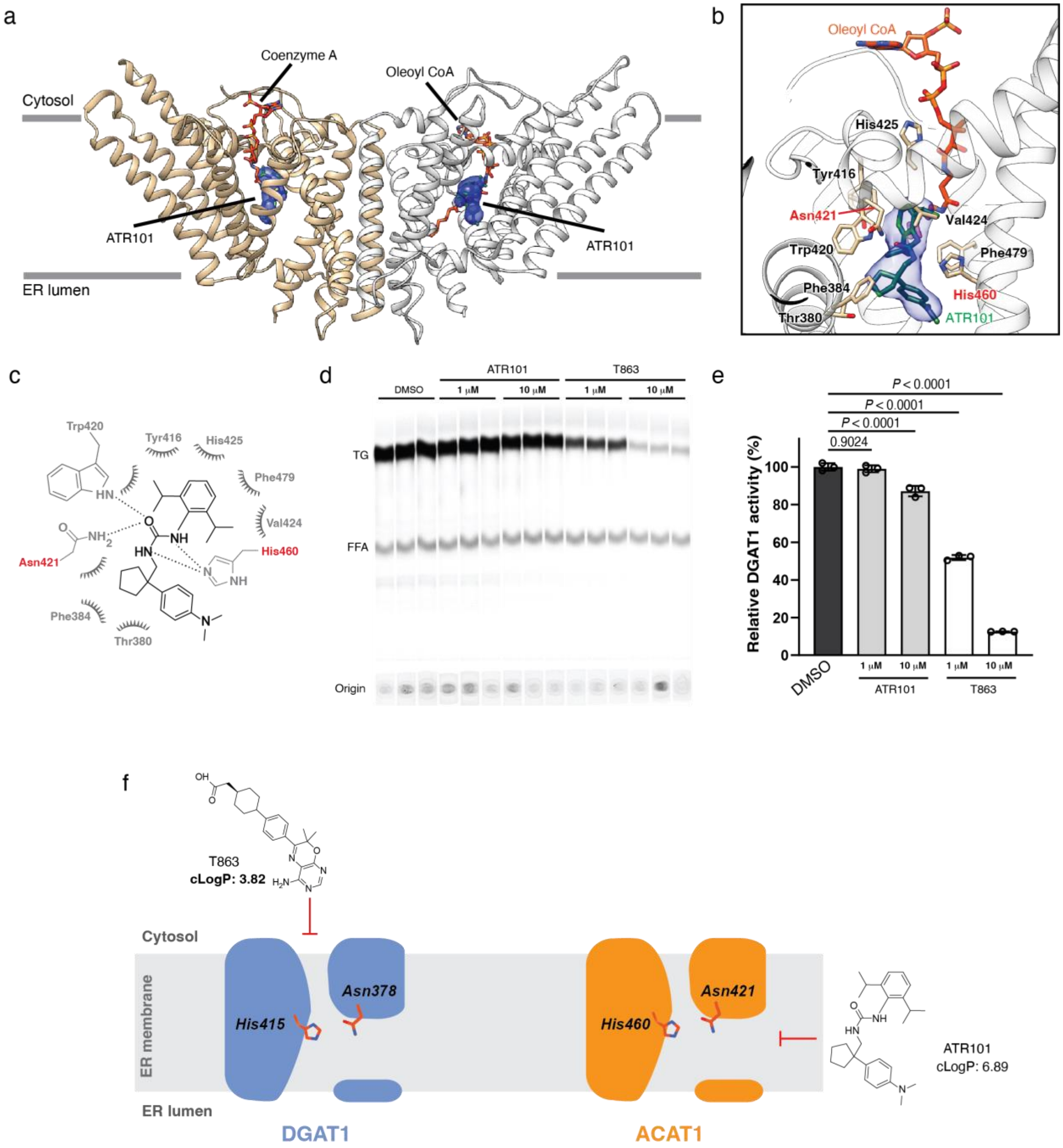

Supplementary Fig. 9 | Cryo-EM structure of human ACAT1 with ATR101. a, Ribbon representation of the published human ACAT1 dimeric structure with the ATR101 inhibitor (EMDB: 21390, PDB: 6VUM)[16]. The ternary complex structure is shown along the membrane plane. In addition to ATR101 (with its EM density depicted in blue), each ACAT1 protomer is bound to oleoyl CoA or coenzyme A. **b**, Zoomed-in view of the ATR101 binding pocket of ACAT1. Residues interacting with ATR101 are shown in orange. The EM density for the bound inhibitor is depicted in blue. **c**, Detailed atomic interactions between ATR101 and ACAT1. Residues participating non-polar interactions with T863 are shown as spiked arcs. **d,e,** ATR101 exhibits low inhibition on human DGAT1. ATR101 exhibits robust inhibition on ACAT1 but minimal inhibition on DGAT1. Experiments were repeated three time with similar results. Data

points are shown as mean ± s.d., calculated from three technical replicates. Statistical analysis by one-way ANOVA tests two-sided pair comparison. FFA, free fatty acid; **f**, Distinct mechanism of action of T863 and ATR101. T863 competes for the fatty acyl CoA binding pocket of DGAT1, and ATR101 likely enters ACAT1 catalytic center through the lateral opening in the ER membrane. The calculated logarithm of the partition coefficient (cLogP) is labeled to denote inhibitor hydrophobicity.

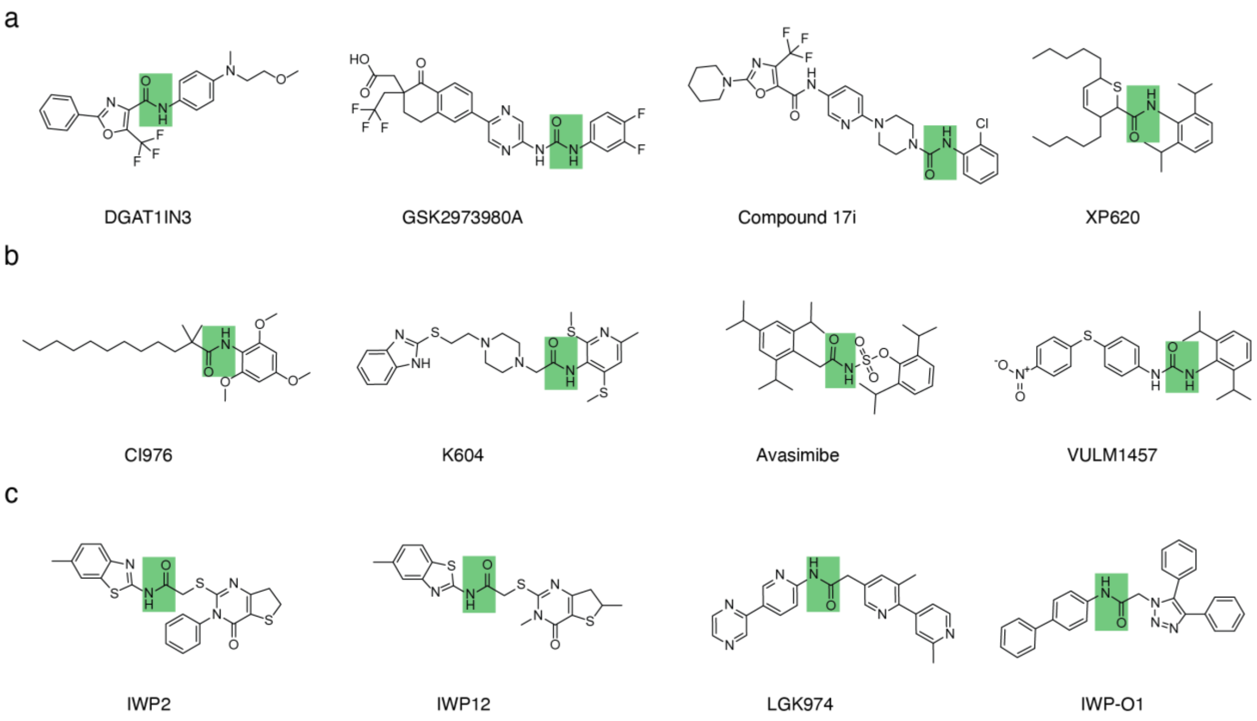

Supplementary Fig. 10 | Examples of amide-containing inhibitors developed on human MBOAT enzymes. Small-molecule inhibitors developed for human DGAT1 (**a**), ACAT1 (**b**), or PROCN (**c**). PROCN mediates palmitoylation of Wnt proteins.

Supplementary Table 1 | Cryo-EM data collection, refinement and validation statistics

	DGAT1-T863<br>EMD-28577, PDB 8ESM		DGAT1-DGAT1IN1<br>EMD-28594, PDB 8ETM
**Data collection and processing**			
Microscope	Talos Arctica	Titan Krios	Titan Krios (3 data sets)
Magnification	45,000	105,000	105,000
Voltage (kV)	200	300	300
Electron exposure (e–/Å^2)	37	44	48/49/50
Defocus range (μm)	1.5-3.5	1.5-3.5	1.0 - 2.5
Pixel size (Å)	0.87	0.83	0.83
Symmetry imposed	C2	C2	C2
Initial particle images (no.)	1,000,870	550,596	143,621/186,726/720,576
Final particle images (no.)	36,885		14,446
Map resolution (Å)	3.2		3.2
FSC threshold	0.143		0.143
Map resolution range (Å)	222.7 - 3.2		212.5 - 3.2
**Refinement**			
Initial model used (PDB code)	6VYI		6VYI
Model resolution (Å)	3.16		3.23
Map sharpening *B* factor (Å^2)	-101		-94
Model composition			
Non-hydrogen atoms	6778		6800
Protein residues	812		812
Ligands	2		2
B factors (Å^2)			
Protein	65.66		77.98
Ligand	44.56		68.73
R.m.s. deviations			
Bond lengths (Å)	0.009		0.008
Bond angles (°)	0.758		0.758
Validation			
MolProbity score	2.08		2.17
Clashscore	9.59		11.49
Poor rotamers (%)	0.56%		0
Ramachandran plot			
Favored (%)	10.95%		11.69%
Allowed (%)	89.05%		88.31%
Disallowed (%)	0		0
